# Increased reliance on top-down information to compensate for reduced bottom-up use of acoustic cues in dyslexia

**DOI:** 10.3758/s13423-021-01996-9

**Published:** 2021-09-24

**Authors:** Hadeer Derawi, Eva Reinisch, Yafit Gabay

**Affiliations:** 1 Department of Special Education, University of Haifa, Mount Carmel, 31905 Haifa, Israel; 2 Disabilities Learning of Study the for Center Research Brain Safra .J Edmond, Vienna, Austria; 3 Sciences of Academy Austrian ,Institute Research Acoustics, Vienna, Austria

**Keywords:** Developmental dyslexia, Dual task, Ganong effect, Lexical bias, Speech perception, Spoken word recognition

## Abstract

Speech recognition is a complex human behavior in the course of which listeners must integrate the detailed phonetic information present in the acoustic signal with their general linguistic knowledge. It is commonly assumed that this process occurs effortlessly for most people, but it is still unclear whether this also holds true in the case of developmental dyslexia (DD), a condition characterized by perceptual deficits. In the present study, we used a dual-task setting to test the assumption that speech recognition is effortful for people with DD. In particular, we tested the Ganong effect (i.e., lexical bias on phoneme identification) while participants performed a secondary task of either low or high cognitive demand. We presumed that reduced efficiency in perceptual processing in DD would manifest in greater modulation in the performance of primary task by cognitive load. Results revealed that this was indeed the case. We found a larger Ganong effect in the DD group under high than under low cognitive load, and this modulation was larger than it was for typically developed (TD) readers. Furthermore, phoneme categorization was less precise in the DD group than in the TD group. These findings suggest that individuals with DD show increased reliance on top-down lexically mediated perception processes, possibly as a compensatory mechanism for reduced efficiency in bottom-up use of acoustic cues. This indicates an imbalance between bottom-up and top-down processes in speech recognition of individuals with DD.

## Introduction

Developmental dyslexia (DD) is one of the most common neurodevelopmental disorders. It is characterized by impaired reading, writing, and spelling skills despite adequate educational opportunities. The most frequent symptoms of DD are phonological in nature but studies suggest a broader range of deficits in the disorder (Démonet et al., 2004). These go beyond the linguistic domain and include impairments in motor skills (Howard Jr et al., 2006; Nicolson & Fawcett, 1994; Stoodley et al., 2006) and temporal processing deficiencies (Farmer & Klein, 1995; Gabay et al., 2019). Domain-specific accounts postulate that DD arises from difficulties in processing (Snowling, 2001) or accessing phonological information (Ramus & Szenkovits, 2008), leading to less distinct phonological representations. Domain-general accounts, in contrast, postulate problems in low-level sensory processing (Goswami, 2011; Tallal, 1984) or procedural learning processes (Nicolson & Fawcett, 2011; Ullman, 2004; Ullman et al., 2020) as an underlying cause of the phonological impairments observed in DD. In particular, a procedural learning impairment may lead to *impaired perceptual category learning* that results in impoverished representations of the phonological characteristics of speech and concomitant difficulties in grapheme–phenome conversion and in learning to read (Gabay & Holt, 2015). Regardless of the cognitive explanation level of DD, there is a consensus among researchers that phonological impairments are among the most common symptoms of DD. Consequently, people with DD are impaired in tasks that require reliance upon phonological representations such as verbal short-term memory, nonword repetition, and rapid naming (see Snowling, 2000, for a review).

Evidence for weaker phonological representations in DD is further supported by the presence of a categorical perception (CP) deficit (Noordenbos & Serniclaes, 2015). In categorical perception tasks, listeners are required to categorize speech sounds that vary along a continuum. Such a task does not elicit a continuous change in perception along the continuum but rather a more or less abrupt switch in perception from one category to another (Goldstone & Hendrickson, 2010). This switch is taken to indicate the perceived phonological category boundary between the sounds. Individuals with DD have been shown to display a less abrupt switch than typically developed (TD) listeners. That is, their categorization functions are shallower, which indicates greater regions of ambiguity at the category boundaries (Godfrey et al., 1981; Mody et al., 1997; Reed, 1989; Tallal, 1980).

Despite this evidence, speech categorization problems in DD have evoked considerable debate among researchers. To date, there is no consensus on whether speech perception impairments in DD are restricted to speech materials or also affect the perception of non-speech sounds (Rosen & Manganari, 2001), as well as whether they occur only for sounds that are cued by temporal information (Vandermosten et al., 2010; Vandermosten et al., 2011). Furthermore, the majority of studies that investigated categorical perception in DD examined speech categorization in isolation (but see Gabay & Holt, 2018, and Gabay et al., 2019). In real-world listening environments listeners have been shown to rely on contextual information to disambiguate speech sounds. In particular, speech recognition in real-world environments involves both bottom-up and top-down processes, that is, use of low-level acoustic as well as higher-level lexical information. For instance, listeners prefer to interpret an ambiguous segment (e.g., along a continuum) within a phonological string in favor of rendering the string as a real word rather than as a nonword. This lexical-bias effect has become known as the Ganong effect (Ganong, 1980). In contrast to categorical perception, where individuals with DD show reduced effects relative to TD listeners, the Ganong effect has been shown to be enhanced in DD, suggesting greater use of top-down information compared to typical listeners (Reed, 1989). This suggestion has been confirmed in different studies (Chiappe et al., 2001; Chiappe et al., 2004; Del Tufo & Myers, 2014). Specifically, it has been suggested that people with DD rely more on top-down processes in order to compensate for the bottom-up sensory-processing deficits.

These findings suggest the possibility that bottom-up speech perception is more effortful in the case of DD, with top-down processes being used as a compensatory mechanism. There are several reasons to believe that this is the case: First, as reviewed above, people with DD show speech perception impairments when required to categorize sounds based on acoustic sensory cues (Noordenbos & Serniclaes, 2015) and use lexical cues in speech recognition to a greater extent than neurotypicals (Reed, 1989). Second, evidence suggests that people with DD differ in their ability to adapt to degraded listening conditions, depending on the availability of top-down information. When trained to adapt to degraded speech signals, typical listeners are able to learn to rely on higher-level top-down information (semantic and lexical knowledge) as well as low-level information (acoustic cues) to better adapt to distorted input (Banai & Lavner, 2012; Guediche et al., 2016). In typical listeners, the learning of distorted speech generalizes across stimuli that share high-level representations (new talker, same tokens) but also to new items that do not share high-level representations with the trained one (same talker, new tokens) (Banai & Lavner, 2012, 2014; Gabay et al., 2017). By contrast, for individuals with DD, such generalization is confined to situations in which trained and untrained information shares the same high-level top-down information (new talker, same tokens) (Gabay et al., 2017) but is not observed in situations in which only low-level sub-lexical cues are shared between the trained and untrained information (same talker, new tokens) (Gabay et al., 2017; Gabay & Holt, 2021). Therefore, it seems that people with DD are capable of adapting to acoustic challenges when utilization of top-down information is possible. Finally, people with DD have difficulty related to implicit utilization of recently presented acoustic information (Ahissar, 2007; Lieder et al., 2019). All these observations support the assumption that bottom-up acoustic sensory processing is less efficient in people with DD than in neurotypicals. In individuals with DD, the ability to use low-level sensory cues is more effortful and therefore they rely more heavily on compensatory mechanisms such as high-level top-down knowledge.

One of the ways to examine whether a process is effortful (resource demanding) is to use a dual-task setting in which participants must perform primary and secondary tasks simultaneously (Navon & Gopher, 1980). The rationale underlying this methodology is based on the assumption that different cognitive processes draw from the same limited pool of cognitive resources (Kahneman, 1973; Posner & Petersen, 1990; Tombu & Jolicœur, 2003). When multiple tasks are executed simultaneously, they can overburden available resources, leading to cognitive interference (the cognitive capacity model; Kahneman, 1973). The more resource demanding the skill of the primary task is, the more likely it is to be affected by dual-task settings. It has been shown that individuals with DD are affected by dual-task settings to a greater extent than neurotypicals (i.e., indicating impaired skill efficiency), but most evidence comes from the motor domain (Bucci et al., 2013; Gabay et al., 2012; Needle et al., 2006; A. Van der Leij & Van Daal, 1999a; Yap & Leij, 1994). Building on the evidence reviewed above, one may speculate that speech recognition is more effortful in people with DD. In order to test this assumption, in the present study we examined the Ganong effect described above, testing individuals with DD and neurotypicals using a dual-task setting.

If speech recognition is more effortful in people with DD, they are more likely to be influenced by dual-task settings compared to neurotypicals. In typical listeners, use of a dual-task setting shifts the balance between top-down and bottom-up processes in speech recognition (Mattys et al., 2014; Mattys & Wiget, 2011). For example, when the Ganong effect is examined in a dual-task setting, typical listeners tend to rely more on top-down information (i.e., greater Ganong effect) than in a single-task setting, presumably due to impaired low-level processing (Mattys & Wiget, 2011; but see Mattys & Scharenborg, 2014). Therefore, both individuals with DD and controls are likely to exhibit a greater Ganong effect under high cognitive load compared to a situation with a low cognitive load. However, if speech recognition is more effortful for those with DD, they could be expected to exhibit a greater modulation of the Ganong effect by cognitive load compared to neurotypicals.

## Methods

### Participants

The sample consisted of 45 university students, of whom 24 were individuals with developmental dyslexia (DD) and 21 were typical readers (TD). All were native speakers of Hebrew, free of neurological disorders, psychiatric disorders, and attention deficits (according to the Adult ADHD Self-Report Scale (ASRS) (Zohar & Konfortes, 2010). Furthermore, all participants had normal or corrected-to-normal vision and hearing. The DD group was recruited mainly through the Yael Learning Disabilities Center at Haifa University in Israel. The presence of a comorbid neurodevelopmental disorder such as attention deficit hyperactivity disorder (ADHD), a specific language impairment (SLI), or any sensory or neurological disability, was an exclusion criterion. The inclusion criteria for the dyslexia group were (1) a formal diagnosis of dyslexia by a qualified psychologist, and (2) a score of at least one standard deviation below the average of the local norms in tests of phonological decoding (non-word reading). Since there are no standardized reading tests for adults in Hebrew, selection was based on local norms, using similar criteria to other studies conducted on Hebrew readers with dyslexia (Gabay et al., 2019; Weiss et al., 2015). Scores of one standard deviation below the mean of the local norms were chosen following the standard practice in the Hebrew literature (Breznitz & Misra, 2003; Shany & Breznitz, 2011). The control group included participants who had no trouble with reading (e.g., at or above the inclusion criteria of the DD group on the nonword-reading test), and were at the same level of cognitive ability (as measured by the Raven testRaven & Court, 1998) as the DD group. The Institutional Review Board at the University of Haifa approved the study, which was conducted in accordance with the Declaration of Helsinki, with written informed consent provided by all participants. Participants received compensation for their participation in the study (120 shekels, approximately $30).

Participants underwent a series of cognitive tests designed to evaluate their cognitive ability (Raven & Court, 1998), verbal short-term memory (Digit span test; Wechsler, 1997), rapid automatized naming skills (RAN tests; Breznitz, 2003), phonological processing skills (phoneme segmentation, phoneme deletion, and Spoonerism), and attentional functions (ASRS; Zohar & Konfortes, 2010). Table 1 presents details of these tasks. Participants' performance in these tests is summarized in Table 2. Results indicate that the groups did not differ in age, attentional or cognitive abilities. However, compared to the control group, the dyslexia group displayed a reading disability profile compatible with the symptomatology of developmental dyslexia. This group differed significantly from the Control group on both rate and accuracy measures of word reading and decoding skills. Moreover, the dyslexia group demonstrated deficits in the three key phonological domains: phonological processing (Spoonerism, phoneme segmentation, phoneme deletion), verbal short-term memory (digit span), and rapid naming (rapid automatized naming).

### Materials

The stimuli included 20 Hebrew words. The words were selected such that half of them began with the sound /s/ as in “sabon” (soap), and half began with /ʃ / as in “shaon” (clock). The second sound in all words was /a/ in order to avoid influences of the quality of the next vowel on the perception of /s/ versus /ʃ/ (Mann & Repp, 1980). All words were of two-syllable length (except for “shauvaa,” which had three syllables) and were stressed on the second syllable. No other tokens of /s/ and /ʃ/ occurred in the words except for the critical initial position. Importantly, the replacement of the initial /s/ or /ʃ/ with the respective other sound did not result in another existing word in Hebrew. All words as well as non-word versions with the initial sounds exchanged were recorded by a male native speaker of Hebrew. The initial sounds of the target words were analyzed acoustically to determine which tokens of /s/ and /ʃ/ were suitable for further manipulation, that is, the creation of an acoustic continuum between /s/ and /ʃ/. To further keep the following context of the critical sounds constant, one token of the vowel /a/ was selected to be used in all words. Criteria for this selection were that the vowel was of approximately average duration of all vowels in second position, and that it was perceived to fit well with the remaining parts of all targets when put back together. In other words, the resulting tokens were to sound natural.

The selected tokens of /s/ and /ʃ/ were then interpolated to a 16-step continuum using a custom-made script in PRAAT (Boersma & Weenink, 2017). That is, each sample of the sounds was mixed to contain a given proportion of signal from each of the two sounds, ranging from 100% /s/ to 100% /ʃ/. This continuum was spliced onto the selected token of the vowel /a/ and then onto the remaining portion of the words, resulting in word-nonword continua such as from /s/abon to /ʃ/abon and nonword-word continua such as from /s/aon-/ʃ/aon (word–nonword). Since all targets had been recorded in their correct form as well as with the initial sounds replaced, for each target the recording of the remaining portion was selected variably from the word and nonword recording such that the whole form sounded more natural. If both forms sounded well, the portion from the recording of the real word was chosen. Based on a pretest and following another experiment using the same stimuli (reported in Gabay, Reinisch, Evan, Binur, & Hadad, under review) a subset of eight continuum steps was selected such that the continua showed no strong overall bias towards any of the endpoints. Importantly, the pretest and previous experiment already indicated that for neurotypical listeners, the stimuli trigger a Ganong effect when no attention to a secondary task was required.

### Visual search task

**Fig. 1:**
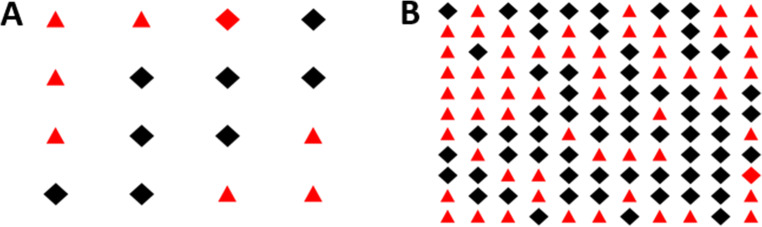
Examples of displays for the visual search task used to manipulate cognitive load (cognitive load). **Panel A:** Visual display used in the low cognitive load condition. **Panel B:** Visual display used in the high cognitive load condition. Both are examples of target-present displays, with the oddball target (red rhombus) in the third column and first row in A and in the eleventh column and ninth row in B

The visual stimuli were adapted from the study of Mattys and Wiget (2011). The grid sizes were chosen based on two brief pilot experiments to ensure that they yield a difference in performance on the secondary task between the high and low cognitive load conditions. Based on these pilots, the visual arrays used in the low cognitive-load condition consisted of grids made of four rows and four columns, resulting in 16 items (see an example in Fig. 1A). The high cognitive-load condition consisted of grids made of 11 rows and 11 columns, resulting in 121 items (see an example in Fig. 1B). The items in each grid were black rhombus and red triangles arranged randomly in the grid. Half the grids contained a red rhombus, which was the oddball target that participants were required to detect. The red rhombus could be anywhere in the grid (see an example in Fig. 1).

## Procedure

The experiment consisted of three sessions. All sessions were conducted in a sound-attenuated booth in front of a 14-in. laptop monitor. Stimuli were presented over Beyer dynamic DT150 headphones at a comfortable listening level that was fixed for all participants (approximately 70 dB SPL). Participants filled out a background questionnaire at home and were invited to complete the linguistic and cognitive battery of tests in the first session. The two cognitive-load conditions were performed as two further separate sessions 1 week apart. Participants were randomly assigned to complete either the low cognitive load or the high cognitive-load condition first. Overall, half the participants performed the high cognitive-load condition in the second session and the low cognitive-load condition in the third session, whereas the other half completed the cognitive-load conditions in the opposite order.

Under both cognitive-load conditions, all combinations of the 20 words with the eight steps of the /s/ to /ʃ/ continuum were presented twice for a total of 320 trials (i.e., 20 words × 8 continuum steps × 2 repetitions) in a different random order for each participant. In each condition, participants were asked to decide whether the first sound of the audio token was /s/ or /ʃ/, regardless of whether it formed an existing word or not. Additionally, they were asked to pay attention to the array displayed on the computer monitor in front of them during the playback of the audio and to search for a red rhombus. The visual array was displayed on the laptop monitor during the playback of the auditory stimulus for a duration of 500 ms, and was immediately followed by two written questions, one about the main task and the other about the visual search task. That is, the first question was "/s/ or /ʃ/?" and participants were instructed to indicate what they had heard by pressing a button on the computer keyboard. Immediately after the participants’ key press, or at the end of a 10-s period, a second written question appeared: "Yes or No?" for the visual search task, where participants had to indicate whether the oddball target (red rhombus) was present. The respective location of the two keys on the keyboard corresponded to the left–right position on the monitor. After key press, or at the end of a 10-s period, there was a 2-s inter-trial interval. The next word was then played, along with the next visual array.

## Results

### Ganong task

**Fig. 2:**
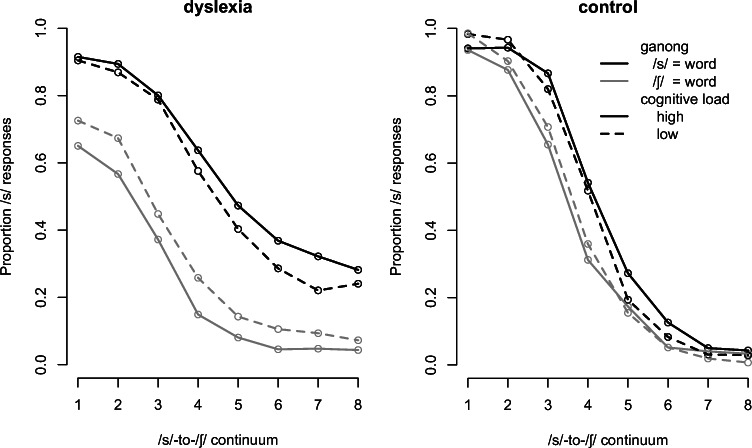
Proportion of /s/ responses over continuum steps for the dyslexia group (**left panel**) and the control group (**right panel**). Colors indicate the word endpoint, with responses to items in which /s/ formed a real word in black and to items in which /ʃ/ formed a real word in grey. Solid lines show responses under high cognitive load and dashed lines show responses under low cognitive load

Statistical analyses were conducted using generalized linear mixed-effects models as implemented in the lme4 package (Bates, Maechler, Bolker, & Walker, 2015) in R (Version 4.0.3, R Core Team, 2020) using a logistic linking function (Jaeger, 2008) to account for the binomial nature of the dependent variable, which was response with /s/ coded as 1 and /ʃ/ coded as 0. Fixed effects were Continuum Step (centered on zero), Lexical Endpoint (whether /s/ or /ʃ/ formed an existing word, coded as 0.5 and -0.5, respectively), Cognitive Load (high load coded as 0.5, low load coded as -0.5), Group (dyslexia coded as 0.5, control coded as -0.5), and all interactions. With this coding, the grand mean was mapped onto the intercept and effects could be interpreted as main effects. The random-effects structure included random intercepts for participants and items (i.e., words) with random slopes for all within-participant factors, that is Continuum Step, Lexical Endpoint, and Cognitive Load over participants. Random slopes over items were not included since they did not improve the model’s fit as assessed by log-likelihood ratio tests. Data and code for the statistics are available at https://osf.io/g4wej/. Table 3 shows the results of this model and Fig. 2 illustrates the effects.

**Table 1:** Psychometric Tests

The following tests were administered according to the test manual instructions:	
1. Raven's Standard Progressive Matrices (Raven, Court, & Raven, 1992). This test is designed to assess nonverbal intelligence. Participants are required to choose an item from the bottom of the figure that would complete the pattern at the top of an image. The maximum raw score for this test is 60. The test reliability coefficient is .9.	
2. Digit Span subtest from the Wechsler Adult Intelligence Scale (WAIS-III; Wechsler, 1997). In this task, participants are required to recall the numbers presented auditorily in the order they were presented by the examiner. The maximum total raw score is 28. Task administration is discontinued after a failure to recall two trials with a similar length of digits. The test reliability coefficient is .9.	
3. Adult ADHD Self-Report Scale (ASRS) measure (Zohar & Konfortes, 2010). An 18-item questionnaire based on the DSM-IV criterion for identifying ADHD in adults. The questions refer to the past 6 months. The ASRS rating scale includes 0–4 rating (very often=5 points, often=4 points, sometimes=3 points, rarely=2 points, never=1 point). A total score of above 51 points is used to identify ADHD.	
4. Rapid Automatized Naming (RAN; Breznitz, 2003): Participants are required to orally name items presented visually as rapidly as possible. The exemplars are drawn from a constant category (RAN colors, RAN categories, RAN numerals, and RAN letters). This requires retrieval of a familiar phonological code for each stimulus and coordination of phonological and visual (color) or orthographic (letters) information quickly on time. The reliability coefficient of these tests ranges from .98 to .99.	
5. One-minute test of words and One-minute test of nonwords (Shatil, 1995a,b). These tests aim to assess reading skills. The one-minute test of words contains nonvowelized words of an equivalent level of complexity. The one-minute test of nonwords contains increasingly complex vowelized nonwords. Each test requires the participant to read aloud as quickly and accurately as possible within one minute. The maximum raw score for the one-minute test of words is 168. The maximum raw score for the one-minute test of nonwords is 86.	
6. Phoneme segmentation test (Breznitz & Misra, 2003): This measure assesses the participant's ability to break a word into its component phonemes. For example, the word *fo* has two phonemes */f/ /o/*. The maximum raw score is 16.	
7. Phoneme deletion test (Breznitz & Misra, 2003): In this test, participants are required to repeat nonwords without a specific phoneme as rapidly as possible. The nonwords are presented auditorily and vary in complexity, with a maximum total raw score of 25.	
8. Spoonerism Test (adapted from Brunswick et al., 1999): Participants are required to switch the first syllables of two word-pairs and then to synthesize the segments to provide new words. The maximum raw score is 12.

Results showed a main effect of Lexical Endpoint, which refers to the Ganong effect, that is, listeners tended to give more /s/ responses if /s/ formed an existing word than if /ʃ/ formed an existing word. However, this main effect was modulated by several interactions. Starting with the highest significant interactions, we found three-way interactions between Lexical Endpoint, Group, and Cognitive Load and between Continuum, Group, and Cognitive Load. The three-way interaction between Continuum, Lexical Endpoint, and Group just failed to reach significance.^1^ Together with the five significant two-way interactions, specifically the two two-way interactions involving the factor Group, they suggest that the effect of Lexical Endpoint was differently impacted by Cognitive Load between groups, as was the effect of Continuum. Specifically, as also visible in Fig. 2, it appears that the effect of Continuum was smaller for the dyslexia group than for the control group (i.e., the slope of the categorization function is shallower) and the effect of Lexical Endpoint was larger for the dyslexia group than for the control group. Moreover, the effect of Lexical Endpoint was distributed differently over the continuum for the two groups.

In order to further inspect the effects of Continuum, Lexical Endpoint, and Cognitive Load as well as their interactions for each Group, two additional mixed-effects models were run on the subsets of data for each group. Models were the same as the model described above minus the fixed-factor Group. Results are shown in Table 4. For the dyslexia group, we found two significant interactions and another that just failed to reach significance. First and foremost, the effect of Lexical Endpoint, that is, the magnitude of the Ganong effect, was modulated by Cognitive Load such that the Ganong effect was larger in the high than in the low cognitive-load condition. Moreover, the interaction of Lexical Endpoint and Continuum suggests that the categorization function was steeper for words that have /ʃ/ as their lexical endpoint than for words with /s/ as their lexical endpoint, and the interaction between Cognitive Load and Continuum suggests that the categorization functions were shallower in the high than low-load condition.

**Table 2:** Demographic and psychometric data of the developmental dyslexia (DD) and control groups

Measurement	Control	Std. Deviation	Dyslexia	Std. Deviation	t value	p
Age (in years)	25.09	2.896	26.04	3.457	0.993	0.326
**Decoding**						
Oral words recognition (accuracy)	114.142	15.634	70.480	19.841	-8.172	0.000
Oral words recognition (speed)	115.523	14.982	74.200	22.561	-7.165	0.000
Oral non-words recognition (accuracy)	62.523	8.829	25.680	10.330	-12.862	0.000
Oral non-words recognition (speed)	66.857	9.717	41.120	13.971	-7.114	0.000
**Naming skills**						
Naming letters (time)	21.904	2.755	25.68	4.288	3.604	0.001
Naming letters (accuracy)	49.952	0.218	49.680	0.690	-1.865	0.072
Naming objects (time)	33.857	5.387	40.680	7.904	3.352	0.002
Naming objects (accuracy)	50.00	0.00	49.680	0.627	-2.551	0.018
Naming numbers (time)	17.714	3.257	21.80	3.135	4.32	0.000
Naming numbers (accuracy)	50.00	0.00	49.48	1.357	-1.915	0.067
**Phonological processing**						
Phoneme segmentation (time)	74.66	16.53	136.04	54.537	5.34	0.000
Phoneme segmentation (accuracy)	15.42	0.676	11.72	3.611	-5.03	0.000
Phoneme deletion (time)	102.0	20.765	190.680	64.070	6.52	0.000
Phoneme deletion (accuracy)	23.19	1.289	18.64	5.154	-3.93	0.000
Spoonerism (time)	115.761	36.006	306.24	171.299	5.419	0.000
Spoonerism (accuracy)	18.857	1.352	14.560	5.284	-3.91	0.000
**Short verbal working memory**						
Digit span	12.04	3.05	10.16	2.47	-2.313	0.025
**Intellectual ability**						
Raven test	72.761	21.881	64.12	27.126	-1.173	0.247
**Attentional functions**						
ASRS	0.37	0.076	0.34	0.090	-1.301	0.200

**Table 3:** Results of the full mixed-effects model

	*b*	SE	*z*	*p*
(Intercept)	-0.94	0.17	-5.57	0.000
Continuum	-1.19	0.07	-17.28	0.000
Lexical Endpoint	1.50	0.23	6.56	0.000
Group	0.26	0.32	0.80	0.424
Cognitive Load	0.13	0.10	1.25	0.212
Continuum: Lexical Endpoint	0.09	0.03	3.41	0.001
Continuum: Group	0.76	0.14	5.51	0.000
Lexical Endpoint: Group	1.29	0.41	3.19	0.001
Continuum: Cognitive Load	0.21	0.03	7.77	0.000
Lexical Endpoint: Cognitive Load	0.54	0.08	6.53	0.000
Group: Cognitive Load	-0.33	0.21	-1.59	0.113
Continuum: Lexical Endpoint: Group	0.10	0.05	1.89	0.059
Continuum: Lexical Endpoint: Cognitive Load	-0.01	0.05	-0.17	0.868
Continuum: Group: Cognitive Load	-0.32	0.05	-5.94	0.000
Lexical Endpoint: Group: Cognitive Load	0.75	0.16	4.53	0.000
Continuum: Lexical Endpoint: Group: Cognitive Load	0.10	0.10	0.99	0.324

**Table 4:** Results for the statistical models, split by group

	*B*	SE	*z*	*p*
Dyslexia group				
(Intercept)	-0.81	0.16	-5.00	0.000
Continuum	-0.81	0.09	-8.75	0.000
Lexical Endpoint	2.16	0.38	5.66	0.000
Cognitive Load	-0.03	0.14	-0.23	0.816
Continuum: Lexical Endpoint	0.15	0.03	5.49	0.000
Continuum: Cognitive Load	0.05	0.03	1.95	0.051
Lexical Endpoint: Cognitive Load	0.92	0.10	8.83	0.000
Continuum: Lexical Endpoint: Cognitive Load	0.04	0.05	0.75	0.455
Control group				
	*B*	SE	*z*	*p*
(Intercept)	-1.06	0.30	-3.54	0.000
Continuum	-1.57	0.10	-15.35	0.000
Lexical Endpoint	0.85	0.16	5.20	0.000
Cognitive Load	0.30	0.15	1.94	0.053
Continuum: Lexical Endpoint	0.04	0.05	0.81	0.416
Continuum: Cognitive Load	0.38	0.05	7.79	0.000
Lexical Endpoint: Cognitive Load	0.17	0.13	1.29	0.197
Continuum: Lexical Endpoint: Cognitive Load	-0.05	0.08	-0.58	0.561

For the control group we found main effects of Continuum (more /s/ responses the lower, that is, the more /s/-like the Continuum Step), Lexical Endpoint (more /s/-responses if /s/ forms a real word), and Cognitive Load (more /s/ responses under high than low cognitive load), with the latter just failing to reach significance. Differences in the regression weights (that relate to effect size) for Continuum and Lexical Endpoint in this model as compared to the model for the DD group reported above highlight the magnitude of group differences for these effects (i.e., smaller effect of Continuum but larger effect of Lexical Endpoint for DD than TD; see Table 4). Note also that unlike the results for the DD group, for the TD group the interaction between Cognitive Load and Lexical Endpoint failed to reach significance, despite the suggestion of a numeric effect in the right direction in the right panel of Fig. 2. This explains the three-way interaction between Group, Cognitive Load, and Lexical Endpoint in the overall analysis. Cognitive Load, however, was involved in a two-way interaction with Continuum such that the categorization function of the Continuum was shallower in the high than in the low-load condition. Looking at the magnitude of this interaction as indicated by the regression weight, one can see that this effect was larger in the control group than in the DD group. This explains the three-way interaction between Group, Cognitive Load, and Continuum in the overall analysis.

### Visual search task

As for performance in the visual search task, we found that the high versus low load manipulation worked. That is, the high-load condition was much more difficult, leading to relatively poorer performance in correctly identifying the presence or absence of the oddball target than the low-load condition. In the high-load condition, the dyslexia group responded about 61% correctly (SD = 49), and the control group 69% (SD = 46). In the low-load condition, the dyslexia group was 94% correct (SD = 24) and the control group 97% correct (SD = 18). These values are similar to those observed by Bosker et al. (2017). As for group differences, a generalized linear mixed-effects model was fit with a logistic linking function, with accuracy (correct = 1, incorrect = 0) as the dependent variable, and the fixed factors group, cognitive load, and their interaction (contrast coded as described above). Random intercepts were fit for Participants and Items, with a random slope for Cognitive Load over Participants. Results confirmed what the mean values suggest. We found main effects of Cognitive Load, such that high load was more difficult than low load (b_(load)_ = -2.72, SE = 0.11, z = -22.8, p < .001), and of Group, such that the dyslexia group performed worse than the control group (b_(group)_ = -0.50, SE = 0.15, z = -3.35, p < .001). The interaction between these factors was not significant (b_(load:group)_ = 0.24, SE = 0.24, z = 1.03, p < .299), likely due to the substantial within-group variability, especially in the high-load condition, which, however, is also similar to previous studies (Bosker et al., 2017).

## Discussion

The present study was designed to test the assumption that speech perception is more effortful in individuals with developmental dyslexia, leading to greater use of top-down information compared to typical readers. For this purpose, we examined the lexical bias effect (i.e., Ganong effect) on phoneme identification in DD and TD readers under dual-task settings. We hypothesized that if speech perception is more effortful in DD, they are more likely to be influenced by a dual-task setting compared to typical listeners. The results confirmed our assumptions. People with DD were more influenced by cognitive load than typical readers. This was manifested in a greater modulation of the Ganong effect (i.e., lexical-bias effect) by load in the DD group compared with the TD group. Specifically, listeners with DD were more inclined to categorize an ambiguous speech sound such that the stimulus could be interpreted as a word rather than non-word, and this effect was greater under high cognitive load compared to low cognitive load. The Ganong effect observed in the TD group was not modulated by load to the same extent, and specifically when analyzing the TD group alone, the interaction between Lexical Endpoint and Cognitive Load failed to reach significance. Note, however, that previous evidence with regard to modulation of the Ganong effect by cognitive load in neurotypical young listeners is mixed. While Mattys and Wiget (2011) were first able to demonstrate the effect, Scharenborg and Mattys et al. (2014) failed to replicate this finding in their young listener group. In the present study the visual search task designed to impose cognitive load was highly successful in taxing central cognitive resources in both DD and TD groups. Listeners' performance on a demanding visual search task was significantly poorer compared to performance in a less demanding visual search task, and this effect did not differ between groups. A lack of taxing processing resources can hence not explain the lack of modulation of the Ganong effect by load in the TD group. Rather, it may be that speech perception is less effortful for typical listeners. Therefore, the load manipulation did not require additional reliance on top-down information under high as opposed to low cognitive load. Notably, the present study compared only conditions of high versus low cognitive load rather than include a condition without cognitive load manipulation because previous studies have already demonstrated an increased Ganong effect in individuals with DD compared to typical readers without added cognitive load (e.g., Reed, 1989). Here we replicated the finding that individuals with DD show a larger lexical bias in speech categorization than do TD listeners (under cognitive load), with the additional novel finding that the Ganong effect is modulated differently by high versus low cognitive load between the two groups.

We observed a greater lexical bias under higher cognitive load in the DD group even at the endpoints of the continuum (Fig. 1). This is likely related to the reduced perceptual acuity in those with DD. Listeners in the control group perceived the continuum endpoints as unambiguous based on acoustic information (the endpoints for the control group in Fig. 1 are close to zero and one). For them lexical information could hence not visibly contribute to categorizing the continuum endpoints. By contrast, listeners in the DD group perceived even the continuum endpoints as somewhat ambiguous, and clearly as less distinct than did neurotypical listeners. Therefore, for DD listeners, lexical information could contribute to phonetic categorization along the entire continuum.

As already noted above, in addition to a greater modulation of the Ganong effect by cognitive load in the DD than TD group, we also observed that listeners with DD found it more difficult than controls to consistently categorize speech sounds along the whole continuum. That is, their categorization functions were shallower than those of controls. Since our /s/-/ʃ/ continuum involves spectral information, this finding is consistent with the notion that speech categorization deficits in people with DD are not restricted only to tasks that involve temporal cues (for a review, see Rosen, 2003). Our study testing phoneme categorization under cognitive load hence corroborates findings without cognitive load manipulations leading to the assumption that phonological representations are not fully differentiated at the phonemic level among listeners with DD (Brady, 1997).

Interestingly this two-way interaction between Group and Continuum, indicating less precise categorization for the DD group, was further modulated by Cognitive Load. Specifically, the categorization function of the continuum was shallower in the high-load condition compared with the low-load condition, yet this effect was larger in the TD group than in the DD group. Such a pattern of results may arise from the fact that the categorization functions of the DD group were already shallower compared to controls, leaving less room for the influence of the cognitive load manipulation. This finding, that the slope of the identification curve is modulated by cognitive load, is consistent with the study of Mattys and Wiget (2011), in which cognitive load led not only to a greater modulation of the Ganong effect (Experiment 1) but also to a reduced ability to discriminate between speech sounds that differed in temporal cues (Voice Onset Time; Experiment 6) (see also, Chiu et al., 2020). Based on their findings, Mattys and Wiget argued that the Ganong effect observed under cognitive load is likely to be a cascaded consequence of impoverished sensory analysis rather than a direct modification of lexical activation by cognitive load.

If greater use of top-down information in speech under cognitive load arises as a consequence of impoverished sensory analysis, then one should observe an increased reliance on that information when sensory analysis is hindered, as in the case of DD. In the present study, people with DD for whom identification curves were shallower compared to neurotypicals, indeed showed a greater reliance on top-down information (greater Ganong effect) and, importantly, such a reliance increased with the cognitive load. Although previous studies reported greater use of top-down information in those with DD (Chiappe et al., 2001; Chiappe et al., 2004; Del Tufo & Myers, 2014; Reed, 1989), our study revealed for the first time a stronger relationship between the amount of available cognitive resources and reliance upon top-down information for recognizing speech in DD compared with TD readers. We observed that the use of top-down information increased as cognitive load increased in the DD group. This may suggest that the compensatory process by which contextual information supports perceptual acuity in those with DD (Reed, 1989) is also responsible for the increased Ganong effect under more demanding listening conditions within the DD group. Such an account would be consistent with previous research suggesting that speech perception deficits in DD are apparent under noisy listening environments (Sperling et al., 2005). Ziegler et al. (2009) argued that when speech recognition in DD is examined under optimal listening conditions, deficient access to certain speech cues might be compensated for by normal access to other redundant speech cues. They found that individuals with DD exhibited a clear speech perception deficit in noise but not in silence. Based on this finding, they argued that the core deficit of DD is a lack of speech robustness in the presence of external or internal noise, suggesting that speech recognition skills in DD are less efficient.

A greater modulation of performance by load in the DD group could also be related to impaired automaticity. In this regard, an influential theory of DD suggests that people with DD have difficulty performing skills automatically (Nicolson et al., 2001; Nicolson & Fawcett, 1990, 2019; Ullman et al., 2020), be it cognitive skills such as reading or motor skills like balance and catching. A consequence of this incomplete automaticity is that dyslexic children need to try harder to compensate even for routine skills that normally achieving children undertake without effort. Declarative knowledge (which includes, among others, lexical and semantic information) has been suggested to play a compensatory role in developmental language disorders, including DD (Hedenius et al., 2013; Ullman & Pullman, 2015). Indeed, evidence suggests that persistent phonological decoding problems in DD may be associated with an increased reliance on whole word memorization for reading (Shaywitz et al., 2008; Van der Leij & Van Daal, 1999). It may therefore be the case that speech recognition based on low-level cues is less automatic and more effortful in DD, and as a consequence, these individuals are more inclined to use top-down information as a compensatory mechanism. Our findings are consistent with such an account that posits that many skills and procedures do not occur automatically in DD (Nicolson et al., 2001; Nicolson & Fawcett, 1990, 2019; Ullman et al., 2020). Support for an automatization deficit in DD is evident mainly in the non-linguistic motor domain (Bucci et al., 2013; Gabay et al., 2012; Needle et al., 2006; Van der Leij & Van Daal, 1999; Yap & Leij, 1994), and the present findings extend previous research into the speech domain. Typical listeners are also influenced by cognitive load, as demonstrated in prior research (Mattys & Wiget, 2011), but if one considers automaticity as a continuum (Logan, 1985), the present findings may suggest reduced automaticity in DD. Specifically, the present findings point to the possibility that in DD there is an imbalance between the ability to use top-down versus bottom-up information in speech recognition. We argue that people with DD are less able to use sensory low-level information efficiently, which leads to greater reliance on top-down information as a compensatory mechanism. This notion is consistent with previous findings in which the ability of dyslexics to generalize speech perceptual learning was intact when trained and untrained information shared high-level top-down information (Gabay et al., 2017) but not when shared information was based only on low-level sub-lexical cues (Gabay et al., 2017; Gabay & Holt, 2021).

One may argue, however, that the greater Ganong effect observed in the DD group reflects a reduced ability to inhibit lexical information rather than an impaired ability to use low-level cues in speech. We judge this possibility as less likely. First, our sample consisted of high-functioning adults with DD. Although previous studies demonstrated reduced inhibition, including lexical inhibition, in DD, as measured by the Stroop task (Brosnan et al., 2002; Everatt et al., 1997), findings were not always consistent (Närhi & Ahonen, 1995; Van der Sluis et al., 2004). In fact, in the study of Beidas et al. (2013), high-functioning adults with DD exhibited better lexical inhibition skills as measured by the Stroop task compared to typical listeners. Therefore, a lexical inhibition deficit is not always apparent in DD, especially when it comes to high-functioning adults. Furthermore, the observation that the DD group exhibited greater use of top-down information alongside impaired speech categorization skills (as evidenced by their shallower categorization functions) supports the possibility that the impaired ability to use bottom-up acoustic sensory analysis leads to greater reliance on top-down information in the DD group.

The present study points to the possibility that speech recognition skills are less efficient in DD compared with typical readers. Notably, speech categorization skills are tuned by the listeners’ linguistic environments through learning (Kuhl, 2004; Meltzoff et al., 2009). It may be the case that impaired low-level perceptual learning limits the ability of people with DD to form precise phonological representations, thus rendering speech recognition skills based on low-level cues less robust. Indeed, recent evidence suggests that such low-level perceptual category learning is significantly disrupted in DD and is associated with their phonological impairments (Gabay & Holt, 2015). The present findings suggest that such a perceptual deficit shifts the balance between bottom-up and top-down processes in speech recognition in DD, leading to greater reliance on the latter as a compensatory mechanism.

## Footnotes

1 Note that when the non-significant four-way interaction is removed from the model, the interaction between Continuum, Lexical Endpoint, and Group becomes significant (p = .03). Nevertheless, we decided against a full model fitting procedure, in which non-significant interactions and fixed factors are removed if they significantly contribute to the model fit. This is because all fixed factors and any of the other interactions are of potential theoretical interest to the interpretation of the study. In the present model, the marginally significant three-way interaction between Continuum, Lexical Endpoint, and Group is in part reflected in the large number of two-way interactions, and is speculated about in the *Discussion*.
